# Variations in the physical demands and technical performance of professional soccer teams over three consecutive seasons

**DOI:** 10.1038/s41598-022-06365-7

**Published:** 2022-02-14

**Authors:** Zeki Akyildiz, Hadi Nobari, Francisco Tomás González-Fernández, Gibson Moreira Praça, Hugo Sarmento, Aytek Hikmet Guler, Esat Kaan Saka, Filipe Manuel Clemente, António J. Figueiredo

**Affiliations:** 1 Sports Science Department, Gazi University, Ankara, Turkey; 2 Department of Exercise Physiology, Faculty of Educational Sciences and Psychology, University of Mohaghegh Ardabili, 56199-11367 Ardabil, Iran; 3 Sports Scientist, Sepahan Football Club, Isfahan, Iran; 4 Department of Physical Activity and Sport Sciences, Pontifical University of Comillas (Centro de Estudios Superiores Alberta Giménez), 07013 Palma, Spain; 5 Sports Department, Universidade Federal de Minas Gerais, Belo Horizonte, Brazil; 6 Research Unit for Sport and Physical Activity, Faculty of Sport Sciences and Physical Education, University of Coimbra, Coimbra, Portugal; 7 Sports Science Faculty, Marmara University, Istanbul, Turkey; 8 Sports Science Faculty, Halıc University, Istanbul, Turkey; 9 Escola Superior Desporto e Lazer, Instituto Politécnico de Viana do Castelo, Rua Escola Industrial e Comercial de Nun’Álvares, 4900-347 Viana do Castelo, Portugal; 10 Instituto de Telecomunicações, Delegação da Covilhã, 1049-001 Lisbon, Portugal; 11 Research Unit for Sport and Physical Activity, Faculty of Sport Sciences and Physical Education, University of Coimbra, Coimbra, Portugal

**Keywords:** Health occupations, Engineering, Mathematics and computing

## Abstract

The purpose of this study was twofold: (i) to analyze the seasonal variations in the physical demands of Turkish Super League teams considering their status in the final rankings and (ii) to analyze the seasonal variations in the technical performance of Turkish Super League teams considering their status in the final rankings. This study followed an observational analytic retrospective design. In the last three seasons of the Turkish Super League (2015–2016, 2016–2017 and 2017–2018), 918 football matches, 54 teams, 25,029 observations were made. The Sentio Sports optical tracking system was used to quantify the physical demands and technical execution of players in all matches. No significant differences of external load were found between seasons analyzed (p > 0.05). The number of lost balls, ball touches in the central corridor, and goals from set pieces increased from season one to the others (p < 0.05), while the number of successful dribbles reduced over time (p < 0.05). As conclusion, it seems not occurred a progressive change in external load over the seasons, while an evolutionary trends regarding technical variables were observed.

## Introduction

Soccer is a dynamic system in which performance is modulated by different dimensions (i.e., physical, physiological, technical, tactical, and psychological)^1,2^. Considering these dimensions, performance analysis has progressively become a consolidated scientific sub-discipline, namely considering specific fields such as match analysis^3,4^ and time-motion analysis^5,6^. Match analyses include analyses of technical skills, tactical behavior, or collective dynamics; time-motion analyses include the physical demands related to the match^7,8^.

Descriptions of soccer team’s performance in different matchs or scenarios using different outcomes in interaction with different moderators have become a popular topic of research^3^. In the case of physical performance, it is relatively well-known that contextual factors such as match location, match status, playing position, and competitive level influence differences and variations in match running^9,10^. Additionally, researchers have tried to identify the evolutionary tendencies of match running demands over seasons in specific matchs^11,12^. As an example, in a longitudinal study conducted in English Premier League teams over seven seasons, a clear increasing tendency for performing sprinting was found, while no meaningful changes were found for total running distance^11^. Similar findings revealing a clear intensification of match running demands in matches were found in another seven-year longitudinal study^12^. In the Spanish league, a study conducted over four consecutive seasons revealed a significant decrease of total distance, while a significant increase in high-intensity running and sprinting was found^2^.

Similar to match running demands, analyses of specific technical outcomes have also been researched in depth, namely to identify main outcomes as passes or finalizations as highly influenced by moderators as contextual factors, the quality of the teams, the quality of the opponents, or playing position^13^. Also, regarding technical performance, interesting research has provided findings about the tendency of evolution over the years in the same competition^11,12,14^. In a study conducted over seven studies comprising 14,700 observations, a clear increasing tendency was found that more passes were made (and that more passes were successful) over time^12^. In a longitudinal study, it was also found that although medium passes progressively increase, the ranking of the teams affects the final classification^11^.

Despite the above-mentioned longitudinal studies’ support for evolutionary tendencies in specific performance outcomes^11,12^, most of studies are conducted in the big five leagues (English, Spanish, French, Italy and German). Further research should be conducted at different competitive levels to confirm whether the evolutionary tendency holds across different countries. Moreover, know possible differences among ranking of the teams may identify how the evolution of physical and technical demands can be related with the classification of the teams and overall quality helping to benchmark the levels expected. Considering that no studies were conducted in the Turkish Super League, it is important to identify and describe the evolutionary tendency of match running performance and technical performance over previous years.

With that in mind, the purpose of this study was twofold: (i) to analyze the seasonal variations in the physical demands of Turkish Super League teams over three consecutive seasons and (ii) to analyze the seasonal variations in the technical performance of Turkish Super League teams over three consecutive seasons.

### Statistical analysis

The present research consisted of the within-participants factor ranking group condition [ranking group 1 (1th, 2th, 3th, 4th, 5th and 6th classified of season 1); ranking group 2 (7th, 8th, 9th, 10th, 11th and 12th classified of season 2; and ranking group 3 (13th,14th,15th,16th,17th and 18th classified of season 3)] and season condition [season 1 (2015–2016); season 2 (2016–2017), and season 3 (2017–2018)]. For the treatment of the data, we use adequate statistical methods to calculate percentages and central and dispersion parameters (arithmetic mean and standard deviation). Data distribution was examined for normality using Kolmogorov–Smirnov test (> 50 samples). A two way, mixed-design ANOVA for ranking group condition and season condition was used to analyze physical and technical performance. Finally, multiple pairwise comparisons were employed for obtaining differences between condition, and the Bonferroni correction was used to compensate the multiple post hoc comparisons. The significance level was set at 5% (p < 0.05). Effect size is indicated with Cohen's d for pairwise comparisons and partial eta squared for Fs. reported. The effect size (*d*) was calculated through Cohen's *d* (23,24). The interpretation of the d regardless of the sign, followed the scale: Very small (0.01), Small (0.20), Medium (0.50), Large (0.80), Very large (1.20), Huge (2.0) as initially suggested by Cohen^15^ and expanded by Sawilowsky^16^. Statistical analyses were performed using SPSS v.26 for Mac (SPSS Inc., Chicago, IL). For all analyses, significance was accepted at p < 0.05.

## Methods

### Study design and experimental approach

This research has a long-term observational research design*.*The fact that it has a long-term research design with big data that investigates the structure of long-term Turkish football increases its importance*.* In Turkey Super League season during 2015–2016, 2016–2017, 2017–2018, while a total of 306 matchs were played in 1 season, on the other hand, a total of 918 matchs were played in 3 seasons. Competition in Turkey Super League were played throughout the 4 days a week. (Friday, Saturday, Sunday and Monday) During the season, each team played a total of 34 matchs and one match per week. 9 matches played every week were recorded by the Sentio Sports optical tracking system. Sentio Sports optical tracking system consists of two cameras with 4 K resolution, a notebook and a Sentio Scope software. Technical parameters and kinematic analysis in these matchs were done automatically by Sentio Scope software. This study was approved by the Ethics Committee of the Halic University (2019/12.11.2019/09–2019/10). The entire study follows the Helsinki Declaration for Humanities.

### Variables

Sentio Scope software automatically tracked the players of both teams during a match and produced the total distance covered by each player, the high intensity running distance covered over 20 km/h, and the sprint distance covered over 24 km/h as csv extension file. The total distance (TD) covered by the team at the end of the match was calculated by taking the sum of all the distance covered by the players of the team during the match. The total high intensity running distance (THID) reached by the team at the end of the match was calculated by taking the sum of the high intensity running distance that the players of the team covered over 20 km/h during the match. The total number of sprints (TS) reached by the team at the end of the match was calculated by taking the sum of the number of players belonging to the team accelerated to over 24 km/h during the match. The total sprint distance (TSD) reached by the team at the end of the match was calculated by taking the sum of the distance the players of the team covered over 24 km/h during the match.

The technical data of all the actions of the players during the match were obtained by the Sentio Scope software over the same system. The technical data obtained are: Playing the ball %, Correct Passing Per Match %, Pass Per Minute, through ball per match, Key pass per match, long pass per match, Passes to the third zone per game, Ball loss per match, Ball win per match, Number of meetings with the ball in the inner hallway, Average goals per game, total goals scored from standing balls, goal from a corner, freekick goal scored, penalty scored, goal scored from throw-in, cross the ball %, Dribbles per match, Successful dribbles per match %. The successful pass percentage (BP) achieved by the team at the end of the match is calculated by proportioning the total number of successful passes made by the players of the team during the match to the total number of passes of the players. The percentage of possession of the ball at the end of the match (TBP) of the team was calculated by dividing the total time that the players owned the ball during the match to the total time of possession of both teams. Necessary permissions have been obtained from Sentio Sports company to publish the work.

### Data collection and measurement

**Figure 1:**
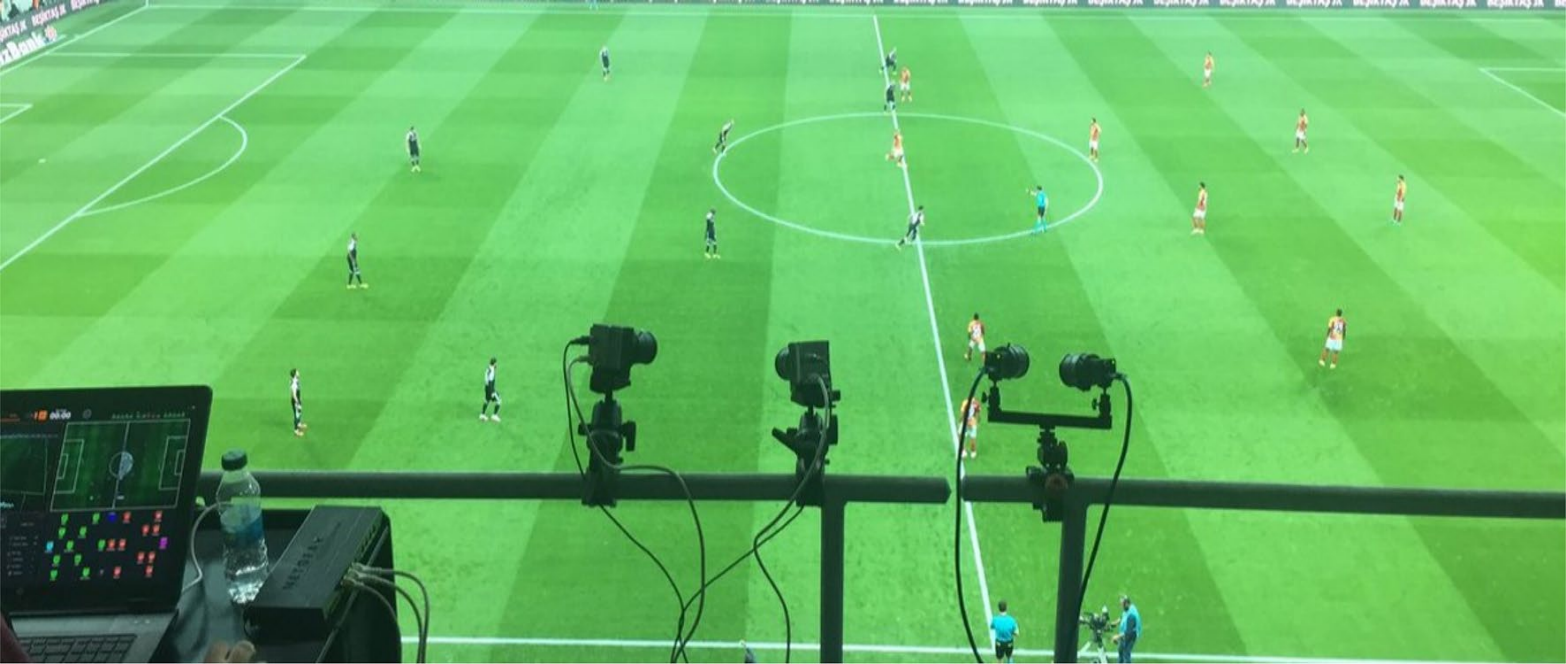
Sentio Sports optical tracking system positioning.

**Figure 2:**
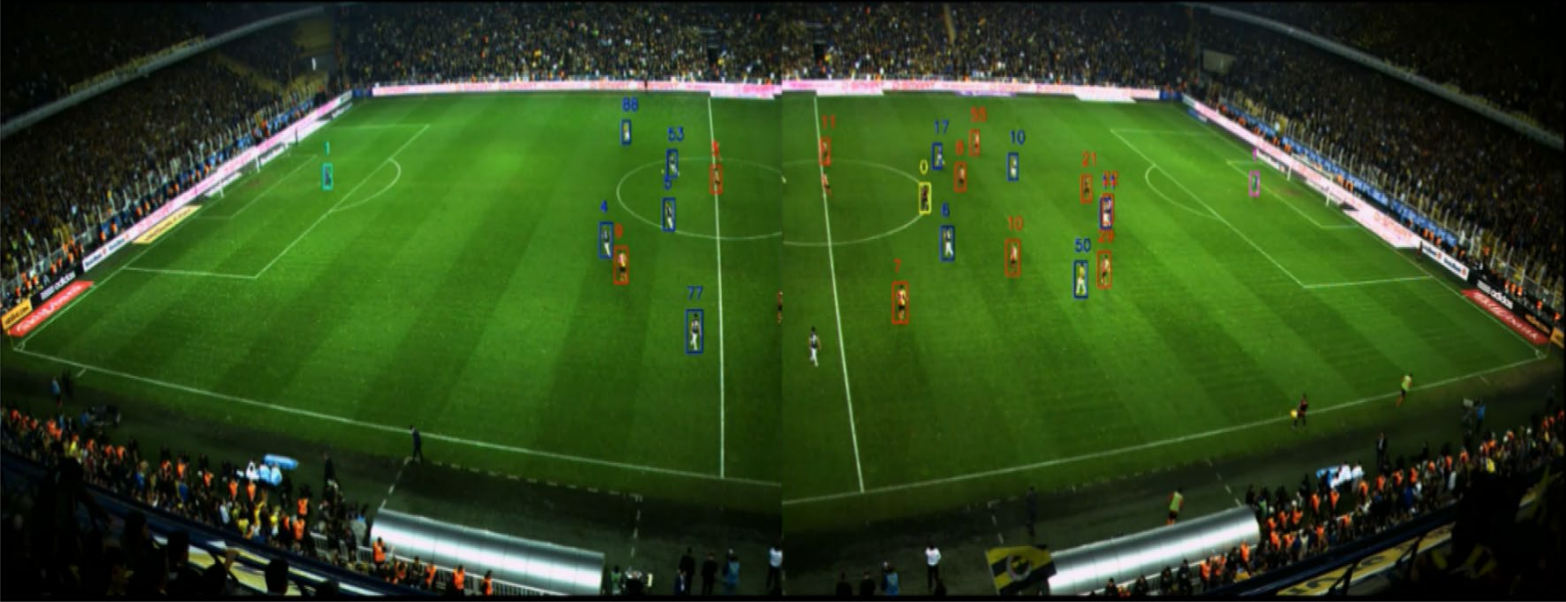
Full field view of cameras belongs to Sentio Sports optical tracking system.

**Figure 3:**
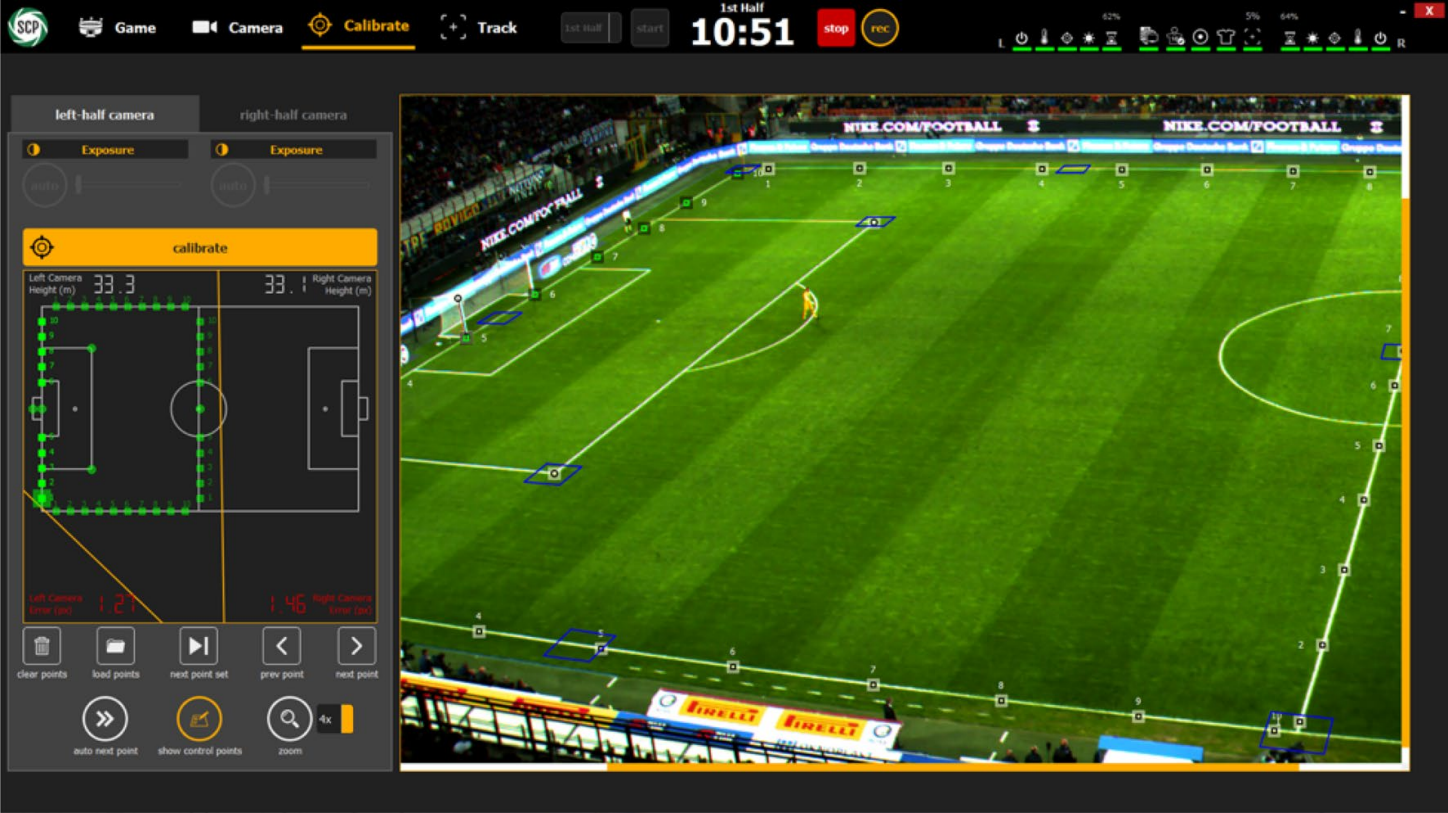
Sentio Sports optical tracking system field calibration.

**Figure 4:**
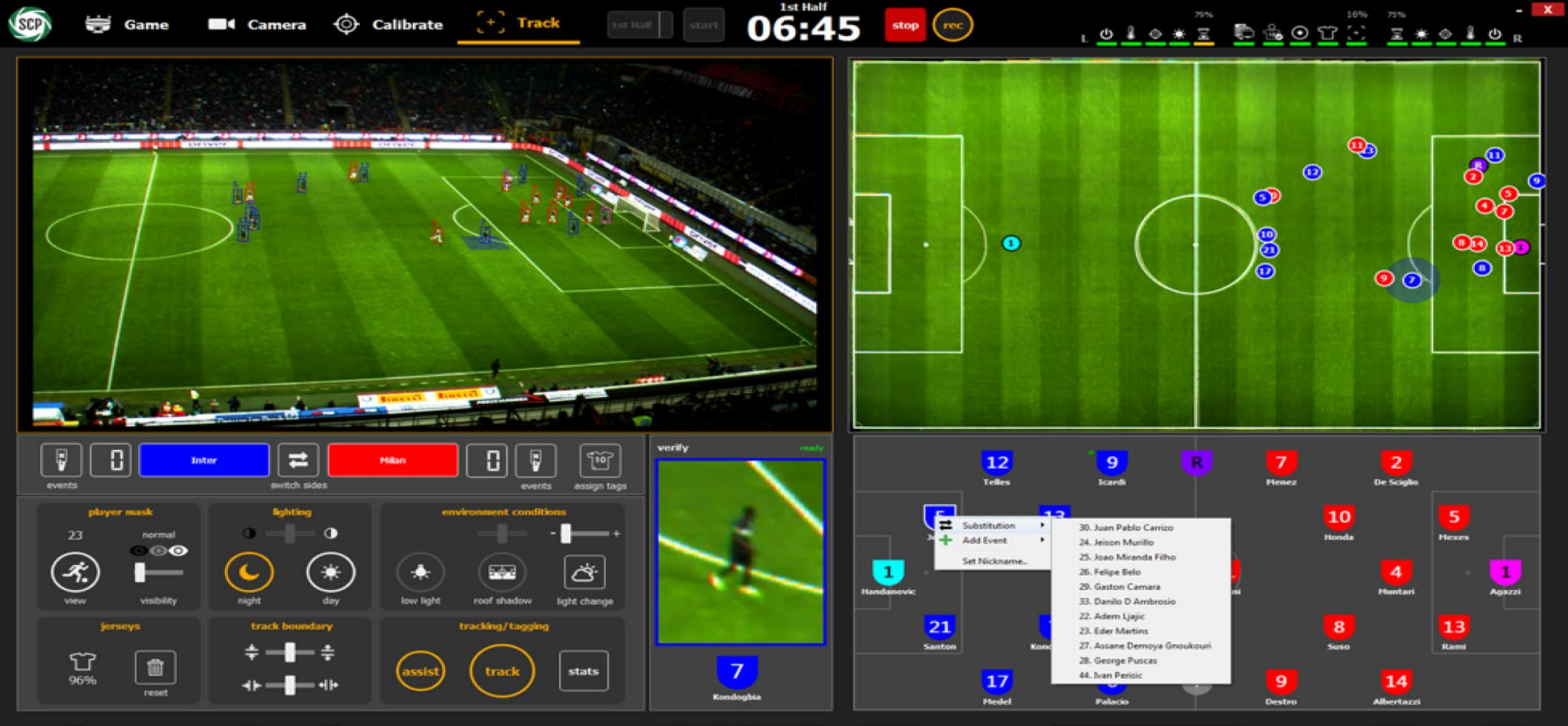
Sentio Sports optical tracking system computer software screen.

Sentio Sports optical tracking system consists of two cameras with 4 K resolution, a notebook and a Sentio Scope software (Fig. 1). This monitoring system has been shown to provide good reliability and accuracy in previous studies^17–19^. The cameras are fixedly positioned in the live broadcast room by the broadcaster, which is at the level of the midfield line, so that they can see the field in two parts (Fig. 2). After the cameras are connected to the computer, the sharpness adjustment and calibration of the cameras on the field image are performed with the Sentio software (Fig. 3). Calibration requires the definition of the number of points requested by the system to the software. After the team staff is encoded into the Scope software by an operator, the system automatically starts tracking the players and recording the location data of the players (Fig. 4). The location data of the match are not assigned to the player by the system since the distances of the players to each other in corner and set ball organizations are too close.Therefore, assigning the registered data of the players confused by the system to the correct player is resolved by the operator's identification of these players, and thus data loss is prevented^20^. Scope software asks questions to the operator to check the locations at regular intervals so that the accuracy of the optical tracking is not reduced during the match.

### League ranking classifications

The league rankings are classified according to Groups: (A) 1.–5. ranking, (B) 6.–10. ranking, (C) 10.–14. ranking and (D) 15th–18th ranking. The league ranking classification is a system created with the performances of the teams during the season. Groups A included the top 4 teams that could qualify for UEFA Champions League (UCL) each season, while Groups B included the next 4 teams around the European qualification. Group B teams were the teams that finished the league after group A. Group C made up the remaining 6 teams that were not challenging for European qualification or battling relegation. Group D consisted of the bottom 6 teams that are typically battling against relegation. The rankings of the teams in the league are based on the points they earn.

### Statistical analysis

The present research consisted of the within-participants factor group condition [group 1 (1,2,3,4,5 and 6 classified of season 1); group 2 (7,8,9,10,11 and 12 classified of season 2; and group 3 (13,14,15,16,17 and 18 classified of season 3)] and season condition [season 1 (2015–2016); season 2 (2016–2017), and season 3 (2017–2018)]. For the treatment of the data, we use adequate statistical methods to calculate percentages and central and dispersion parameters (arithmetic mean and standard deviation). Analyses of variance (ANOVAs) were used to analyze physical [(i) TD; (ii) THID; (iii) TS; and (iv) TSD], and technical performance [(i) playing the ball%; (ii) correct passing per match %; (iii) pass per minute; (iv) through ball per match; (v) key pass per match; (vi) long pass per match; (vii) passes to third zone per match; (viii) ball loss per match; (ix) ball win per match, (x) number of meeting with the ball in the inner hallway; (xi) average goals per match; (xii) total goals scored from standing balls; (xiii) goal from a corner; (xiv) frekick goal scored; (xv) penalty scored; (xvi) goal scored form throw-in (were not performed, finally there were not enough variance for do the analysis); (xvii) cross the ball %; (xviii) dribbling per match; (ixx) dribbling per match %].

The Sphericity assumed were used to compare individual data points obtained from each season. Statistically significant effects were further analyzed by paired-sample t-tests [0.2 (small); 0.5 (medium) and > 0.8 (large)] corrected by Holm-Bonferroni for pairwise comparisons. Effect size is indicated with Cohen's d for pairwise comparisons and partial eta squared for Fs. reported. Data were analyzed using software Statistica (version 10.0; Statsoft, Inc., Tulsa, OK, USA). For all analyses, significance was accepted at p < 0.05.

### Ethics approval and consent to participate

The study fully adheres to the ethical principles of the declaration of Helsinki as wel as GCP guidelines. The ethics committee for clinical research at the Haliç University approved this study. Informed consent was obtained from all subjects (Number:2019/12.11.2019/09-2019/10).

### Consent for publication

Permission was obtained from the company providing the data for publication.

## Results

### Physical performance

**Figure 5:**
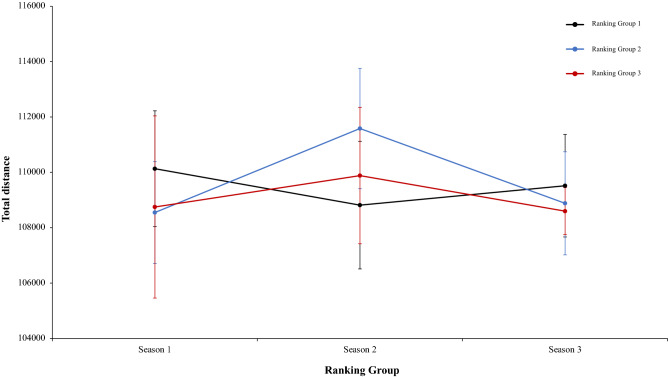
Physical performance. Mean TD (± SE) as a funtion of Ranking and Season Condition.

**Figure 6:**
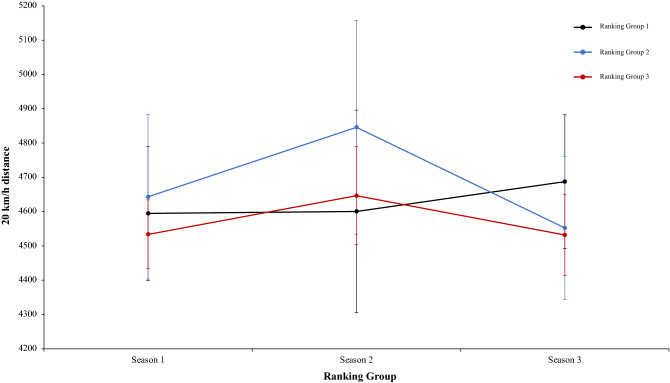
Physical performance. Mean THID (± SE) as a funtion of Ranking and Season Condition.

**Figure 7:**
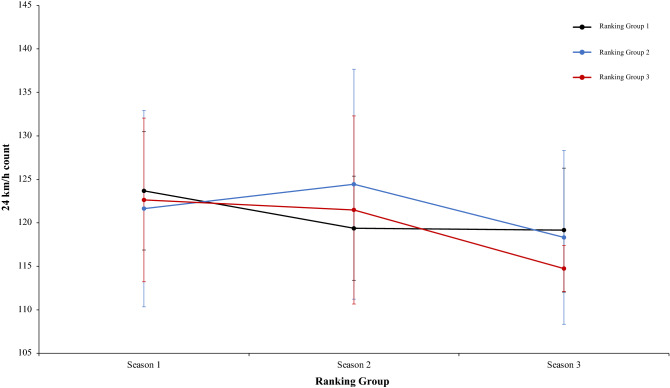
Physical performance. Mean TS (± SE) as a funtion of Ranking and Season Condition.

**Figure 8:**
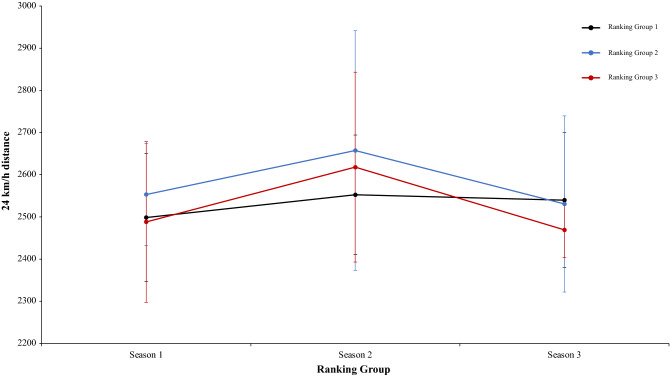
Physical performance. Mean TSD (± SE) as a funtion of Ranking and Season Condition.

A two-way mixed ANOVA with ranking group means TD, did not reveal a significant main effect of season condition, F (2.4) = 1.35, p = 0.26, η^2^ = 0.28, and the interaction between season condition and group, F (2.4) = 1.68, p = 0.17, η^2^ = 0.13. The main effect of ranking group condition neither was significant, *F* < 1 (Fig. 5). In a second two-way mixed ANOVA with ranking group means THID, did not show a significant main effect of season condition, F (2.4) = 1.52, p = 0.22, η^2^ = 0.38, and neither an interaction between season condition and ranking tears, F (2.4) = 1.20, p = 0.32, η^2^ = 0.09. The main effect of ranking group condition was in the same line and did not show significant results, *F* < 1 (Fig. 6). Another two-way mixed ANOVA with ranking group means TS, did not reveal significant main effect of season condition, F (2.4) = 1.70, p = 0.19, η^2^ = 0.69. The effect of ranking group condition and interaction between ranking group and season neither were significant, *F* < 1 in all cases (Fig. 7). Lastly, other two-way mixed ANOVA with ranking group means TSD, did not expose a significant main effect of season condition, F (2.4) = 1.66, p = 0.20, η^2^ = 0.77. The main effect of ranking group condition neither was significative, F (2.4) = 2.07, p = 0.24, η^2^ = 0.50. In addition, the interaction between ranking group and season did not reveal significant effects, *F* < 1 (Fig. 8). Physical data of the player during the match [TD, THID, TS and TSD (SE)] as a function of ranking group and season condition are shown Table 1.

**Table 1:** Physical data of the player during the match [TD, THID, TS and TSD (SE)] as a function of Ranking group and Season Condition.

%N = 18 teams															
Season 1						Season 2					Season 3				
R	T	TD	THID	TS	TSD	T	TD	THID	TS	TSD	T	TD	THID	TS	TSD
**Ranking group 1**															
1	B	108,700	4370.35	130.32	2385.74	B	109,100	4324.56	109.74	2330.82	G	109,900	4775.73	129.12	2790.43
2	F	109,100	4694.16	128.00	2444.95	Ba	110,800	4689.81	124.24	2668.80	F	110,300	4694.16	111.44	2444.95
3	Ko	113,300	4602.66	112.35	2382.17	F	106,000	4281.65	117.44	2506.09	Ba	111,900	4782.54	118.26	2465.81
4	Ba	111,600	4782.54	123.03	2465.81	G	110,400	4668.79	119.62	2503.29	B	106,500	4370.35	112.85	2385.74
5	Os	110,500	4343.75	128.65	2522.19	An	105,900	4544.89	118.26	2572.41	T	108,400	4931.02	126.24	2685.81
6	G	107,600	4775.73	119.74	2790.43	T	110,700	5093.59	126.97	2733.29	Gö	110,100	4570.77	117.09	2466.52
		110,133.33 ± 2090.61	4594.86 ± 178.57	123.68 ± 6.22	2498.55 ± 139.03		108,816.67 ± 2302.53	4600.55 ± 269.78	119.38 ± 5.47	2552.45 ± 129.31		109,516,67 ± 1850.86	4687.43 ± 178.13	119.17 ± 6.50	2539.87 ± 145.89
**Ranking group 2**															
7	K	110,400	4934.10	141.24	2703.10	A	110,500	4559.33	109.06	2340.77	S	107,400	4474.99	116.71	2493.66
8	A	109,000	438.85	109.38	2544.37	Ge	112,000	5000.43	128.62	2797.23	K	112,100	4934.10	124.09	2703.10
9	An	105,300	4463.48	113.26	2413.69	Ko	114,200	4669.30	111.44	2352.31	Ka	108,700	4652.55	131.74	2732.17
10	Ge	107,800	4647.23	126.68	2438.06	K	110,700	4930.23	121.41	2576.08	M	108,800	4414.22	101.62	2149.57
11	Bu	110,000	4496.89	119.53	2534.41	Kar	113,700	5355.30	144.00	3052.05	A	109,500	4385.85	116.41	2544.37
12	T	108,800	4931.02	119.74	2685.81	Al	108,400	4560.54	132.12	2825.31	Al	106,800	4452.85	119.44	2561.41
		108,550.00 ± 1839.29	4643.09 ± 218.87	121.64 ± 10.31	2553.24 ± 110.46		111,583.33 ± 2172.02	4845.86 ± 284.38	124.44 ± 12.07	2657.29 ± 259.31		108,883,33 ± 1860.56	4552.42 ± 190.81	118.33 ± 9.13	2530.71 ± 190.64
**Ranking group 3**															
13	R	111,100	4652.55	133.68	2732.17	O	109,100	4754.57	124.21	2788.26	Bu	109,000	4496.89	115.74	2534.41
14	Ga	104,900	4474.99	117.56	2493.66	B	112,600	4747.47	109.76	2337.23	An	108,200	4463.48	114.85	2413.69
15	Ka	104,400	4452.85	113.71	2561.41	Ka	106,900	4587.15	132.94	2781.44	Ko	107,600	4602.66	113.18	2382.17
16	S	110,700	4414.22	129.38	2149.57	R	112,700	4769.32	134.91	2877.82	O	107,800	4343.75	113.44	2522.19
17	E	112,000	4570.77	129.79	2466.52	Ga	107,600	4399.23	114.59	2493.06	Ge	109,400	4647.23	111.88	2438.06
18	M	109,400	4637.39	111.74	2525.39	Ad	110,400	4620.78	112.50	2429.86	Kar	109,600	4637.39	119.38	2525.39
		108,750.00 ± 3288.01	4533.79 ± 91.75	122.64 ± 8.59	2488.12 ± 173.89		109,883.33 ± 2462.05	4646.42 ± 130.57	121.49 ± 9.87	2617.94 ± 205.36		108,600.00 ± 848.53	4531.90 ± 108.58	114.75 ± 2.41	2469.32 ± 60.34

Notes: Data are presented as means and standard deviations. TD: Total Distance; THID: Total high intensity running distance; TS: Total number of sprints; TSD: Total sprint distance; R: Ranking; Teams: Beşiktaş (B); Fenerbahçe (F); Konyasport (Ko); Başakşehir (Ba); Osmanlıspor (O); Galatasaray (G); Kasımpaşa (K); Akhisar (A); Antalyaspor (An); Gençlerbirliği (Ge); Bursaspor (Bu); Trabzonspor (T); Rizespor (R); Gaziantepspor (Ga); Kayserispor (Ka); Sivasspor (S); Eskişehirspor (E); Mersin İY (M); Karabükspor (Kar); Alanyaspor (Al); Adanaspor (Ad), Göztepe (Gö), and Malatyaspor (M.

### Technical performance

**Figure 9:**
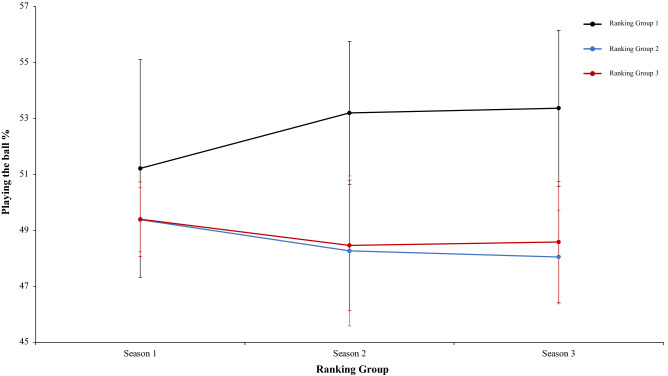
Technical performance. Mean playing the ball percentage (± SE) as a funtion of Group and Season Condition.

**Figure 10:**
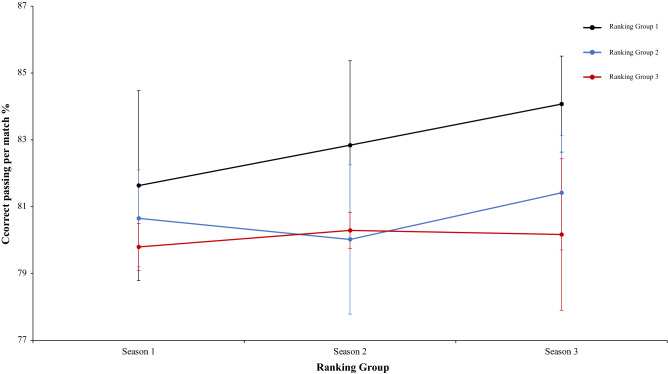
Technical performance. Mean correct passing per match percentage (± SE) as a funtion of Ranking and Season Condition.

**Figure 11:**
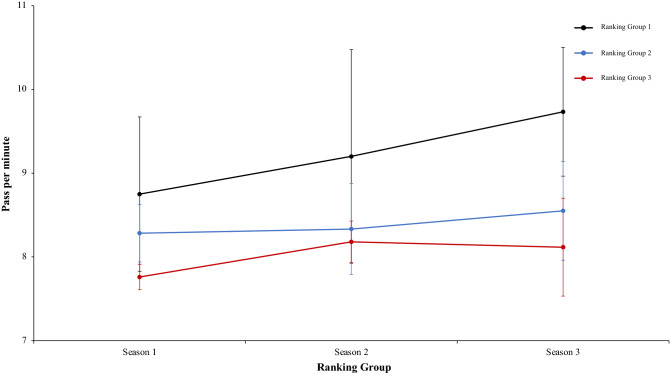
Technical performance. Mean correct pass per minute (± SE) as a funtion of Ranking and Season Condition.

**Figure 12:**
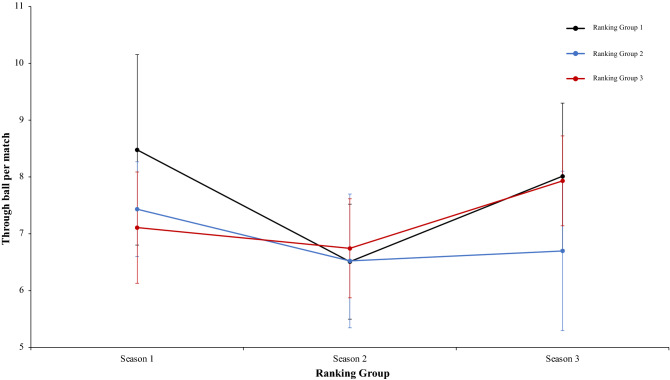
Technical performance. Mean throught ball per match (± SE) as a funtion of Ranking and Season Condition.

A two-way mixed ANOVA with ranking group means playing the ball percentage, revealed a significant main effect of ranking group condition, F (2.4) = 13.88, p = 0.01, η^2^ = 0.87, teams of ranking 1 have a playing the ball percentage generally higher than ranking group 2 or 3. On the one hand, the effect of season condition was not significant, *F* < 1. On the other hand, interaction between ranking group and season condition showed no significant differences, F (2.4) = 1.12, p = 0.35, η^2^ = 0.09. In a reference to main effect of ranking group condition, pairwise comparisons showed significant differences between the ranking group 1 and ranking group 2, *t*(17) = 4.29, *p* < 0.001, d = 1.58, and between the ranking group 1 and the ranking group 3, *t*(17) = 3.62, *p* < 0.002, d = 1,50. The comparison between ranking group 2 and ranking group 3, *t*(17) = − 0.34, *p* < 0.073, d = − 0.05, failed to reach statistical significance (Fig. 9). Moreover, a two-way mixed ANOVA with ranking group means correct passing per match percentage, showed a significant effect of ranking group condition, F (2.4) = 11.88, p = 0.002, η^2^ = 0.85. Teams of ranking group 1 performed correct passing per match percentage with more efficacy than the teams of ranking group 2 and ranking group 3. In fact, pairwise comparisons showed significant differences between the ranking group 1 and the ranking group 2, *t*(17) = 3.51 *p* < 0.002, d = 1.05, and between the ranking group 1 and ranking group 3, *t*(17) = 4.44, *p* < 0.001, d = 1.53. The comparison between ranking group 2 and ranking group 3, *t*(17) = 1.70, *p* < 0.010, d = − 0.40, was not significative. The effect of season condition was not significative, F (2.4) = 1.69, p = 0.19, η^2^ = 0.50, and the interaction between ranking group and season condition neither showed significative data, *F* < 1 (Fig. 10). Furthermore, a two-way mixed ANOVA with ranking group means correct pass per minute, showed a significant effect of ranking group condition, F (2.4) = 19.54, p = 0.001, η^2^ = 0.90. Teams of ranking group 1 realized significative more pass per minute than ranking group 2 and 3. In addition, ranking group 2 performed also significative more pass per minute than ranking group 3. In fact, a pairwise comparisons showed significant differences between the ranking group 1 and the ranking group 2, *t*(17) = 3.53, p = 0.002, d = 1.51, and between the ranking group 1 and the ranking group 3, *t*(16) = 6.15, p = 0.001, d = 0.93. Furthermore, the comparison between ranking group 2 and ranking group 3, *t*(16) = 3.18, p = 0.006, d = 0.53, also was significative. The effect of season condition and interaction between ranking group and season were not significant, F (2.4) = 2.43, p = 0.09 η^2^ = 0.63, and *F* < 1 respectively (Fig. 11). In the same line of above analysis, another two-way mixed ANOVA with ranking group means through ball per match not revealed significative main effects on ranking group condition and neither in the interaction between ranking group and season condition, F (2.4) = 1.49, p = 0.32, η^2^ = 0.42, and F (2.4) = 1.38, p = 0.25, η^2^ = 0.10, respectively. Interestingly, data revealed a significant main effect of season condition, F (2.4) = 4.75, p = 0.01, η^2^ = 0.63. Teams of ranking group 1 performed through ball per match with more effectiveness than the teams of ranking group 2 and ranking group 3. In fact, pairwise comparisons showed significant differences between the ranking group 1 and the ranking group 2, *t*(17) = 1.87, p = 0.07, d = 2.81, and between the ranking group 1 and ranking group 3, *t*(17) = 1.08, p = 0.35, d = 1.44. The comparison between ranking group 2 and ranking group 3, *t*(17) = 1.73, p = 0.29, d = − 1.68, was not significative (Fig. 12). Technical data of the player during the match [Playing the ball %, Correct Passing per Match %, Pass per minute; Through ball per match (mean and SE)] as a function of ranking group and season condition are shown Table 2.

**Table 2:** Technical data of the player during the match [Playing the ball %, Correct Passing per Match %, Pass per minute; Through ball per match (mean and SE)] as a function of Ranking group and Season Condition.

Season 1						Season 2					Season 3				
R	T	Playing the ball %	Correct passing per match %	Pass per minute	Through ball per match	T	Playing the ball %	Correct passing per match %	Pass per minute	Through ball per match	T	Playing the ball %	Correct passing per match %	Pass per minute	Through ball per match
**Ranking group 1**															
1	B	55.5	84.19	9.6	8.92	B	55.7	85.91	10.5	7.57	G	53.9	84.33	9.8	8.28
2	F	54.8	81.92	8.3	8.92	Ba	51.1	81.8	9.3	5.5	F	52	84.12	10	6.16
3	Ko	47.7	80.54	8.8	8.35	F	53	81.9	8.5	6.85	Ba	55.7	85.9	10.8	8.06
4	Ba	49.7	80.67	8.3	5.4	G	56.9	86.04	10.9	7.71	B	56.7	85.24	10.1	7.71
5	Os	46.2	77.28	7.5	10.5	An	50.7	79.95	7.7	5.45	T	53	82.35	8.7	10.16
6	G	53.4	85.19	10	8.77	T	51.8	81.42	8.3	5.97	Gö	48.9	82.47	9	7.7
		51.22 ± 3.89	81.63 ± 2.84	8.75 ± 0.92	8.48 ± 1.68		53.20 ± 2.55	82.84 ± 2.53	9,20 ± 1.28	6.51 ± 1.01		53.37 ± 1.45	84.07 ± 1.44	9.73 ± 0.77	8.01 ± 1.29
**Ranking group 2**															
7	K	50.1	79.84	7.9	6.56	A	43.8	77.1	8.2	6.25	S	48.1	80.59	9.1	6.94
8	A	47.9	78.71	8.5	7.08	Ge	47.4	78.78	7.5	7.44	K	47.2	79.92	9	5.12
9	An	49.7	81.94	8.5	8.61	Ko	49.4	81.18	9	7.79	Ka	51.3	80.43	9	6.77
10	Ge	48.2	79.87	7.8	8.2	K	48	82.87	8.7	4.8	M	46.7	79.58	8.3	5.25
11	Bu	49.5	80.97	8.4	6.65	Kar	51.9	78.45	8	7.27	A	47.9	–	8.3	8.93
12	T	50.9	82.57	8.6	7.49	Al	49.1	81.73	8.6	5.58	Al	47.1	80.93	7.6	7.18
		49.38 ± 2.55	80.65 ± 1.45	8.28 ± 0.34	7.43 ± 0.83		48.27 ± 2.68	80.02 ± 2.23	8.33 ± 0.54	6.52 ± 1.18		48.05 ± 2.47	80.29 ± 1.71	8.55 ± 0.59	6.70 ± 1.40
**Ranking group 3**															
13	R	48.1	84.33	7.7	6.45	O	51,7	82.32	8	7.86	Bu	47.5	80.82	8.6	6.7
14	Ga	–	84.12	–	–	B	47.2	82.68	8	5.21	An	51.3	82.14	8.4	7.37
15	Ka	49.1	85.9	7.6	5.51	Ka	49.1	82.39	8	6.77	Ko	48.6	81.84	8.7	8.4
16	S	51.7	85.24	7.8	7.66	R	45	82.6	8.5	6.88	O	48.5	79.4	7.5	8.94
17	E	49.3	82.35	7.7	7.16	Ga	–	79.72	–	–	Ge	45.2	75.98	7.3	8
18	M	48.2	82.47	8	8.2	Ad	47.8	78.78	8.4	7.15	Kar	50.4	80.82	8.2	8.17
		49.28 ± 2.79	84.07 ± 0.70	7.76 ± 0.15	7.00 ± 1.05		48.16 ± 2.47	81.42 ± 0.54	8.18 ± 0.25	6.77 ± 0.97		48.58 ± 2.16	80.17 ± 2.27	8.12 ± 0.58	7.93 ± 0.79

Data are presented as means and standard deviations. TD: Total Distance; THID: Total high intensity running distance; TS: Total number of sprints; TSD: Total sprint distance; R: Ranking; Teams: Beşiktaş (B); Fenerbahçe (F); Konyasport (Ko); Başakşehir (Ba); Osmanlıspor (O); Galatasaray (G); Kasımpaşa (K); Akhisar (A); Antalyaspor (An); Gençlerbirliği (Ge); Bursaspor (Bu); Trabzonspor (T); Rizespor (R); Gaziantepspor (Ga); Kayserispor (Ka); Sivasspor (S); Eskişehirspor (E); Mersin İY (M); Karabükspor (Kar); Alanyaspor (Al); Adanaspor (Ad), Göztepe (Gö), and Malatyaspor (M).

**Figure 13:**
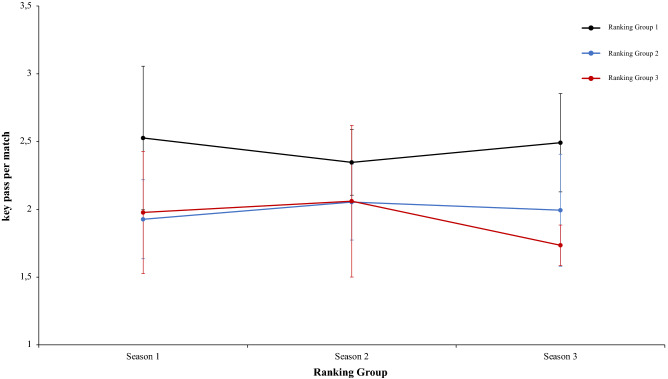
Technical performance. Mean key pass per match (± SE) as a funtion of Ranking and Season Condition.

**Figure 14:**
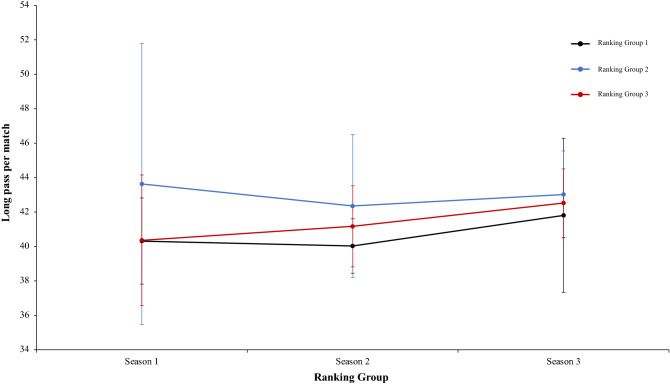
Technical performance. Mean long pass per match (± SE) as a funtion of Ranking and Season Condition.

**Figure 15:**
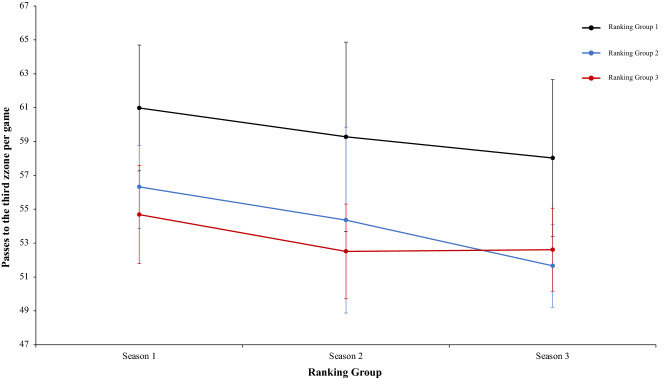
Technical performance. Mean passes to third zone (± SE) as a funtion of Ranking and Season Condition.

**Figure 16:**
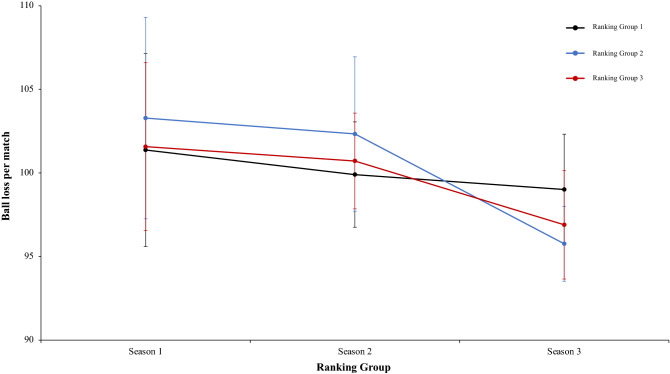
Technical performance. Mean ball loss per match (± SE) as a funtion of Ranking and Season Condition.

In this connection, a two-way mixed ANOVA with ranking group means key pass per match revealed a significant main effect of ranking group condition, F (2.4) = 13.97, p = 0.01, η^2^ = 0.87. Teams of ranking group 1 performed more key pass per match than ranking group 2 and ranking group 3. In fact, pairwise comparisons showed significant differences between the ranking group 1 and the ranking group 2, *t*(17) = 3.75, p = 0.001, d = 4.18, and between the ranking group 1 and ranking group 3, *t*(17) = 3.89, p = 0.001, d = 4.96. The comparison between ranking group 2 and ranking group 3, *t*(17) = 0.42, p = 0.67, d = 1.26, was not significative (Fig. 12). Continuing with the previous analysis, the interaction between ranking group and season and the main effect of season did not reveal significant data, *F* < 1, in both cases (Fig. 13). In the same way, other two-way mixed ANOVA with ranking group means long pass per match revealed significative effect of ranking group condition, F (2.4) = 7.15, p = 0.04, η = 0.78. Teams of ranking group 2 performed with more effectiveness key pass per match than ranking group 3 and ranking group 1. In fact, pairwise comparisons did not showed significant differences between the different ranking: ranking group 1 vs ranking group 2, ranking group 1 vs ranking group 3, ranking group 2 vs ranking group 3, *t*(17) =  − 1.45, p = 0.15, d = − 0.53, *t*(17) =  − 0.63, p = 0.53, d = − 0.21, and *t*(17) = 1.28, p = 0.21, d = 0.39, respectively. Last, no significant effects were identified in season condition and neither in the interaction between ranking group and season was not significant, *F* < 1 (Fig. 14). A new two-way mixed ANOVA with ranking group means passes to third zone per match, revealed a significant main effect of ranking group condition, F (2.4) = 53.62, p = 0.001, η^2^ = 0.96. Teams of ranking group 1 act more effectively and executed more passes to the third zone per game than ranking group 2 or 3. The effect of season condition also revealed a significant main effect of season, F (2.4) = 3.27, p = 0.04, η^2^ = 0.86. At the end, the interaction between ranking group and season condition was not significant, F (2.4) = 0.25, p = 0.90, η^2^ = 0.02. On the one hand, regarding to main effect of ranking group condition, pairwise comparisons showed significant differences between the ranking group 1 and ranking group 2, *t*(17) = 3.62, p = 0.002, d = 1.29, and between the ranking group 1 and the ranking group 3, *t*(17) = 5.01, p = 0.001, d = 1.66. The comparison between ranking group 1 and ranking group 2, *t*(17) = 0.81, p = 0.042, d = 0.32, did not show statistical significance. On the other hand, regarding to main effect of season condition, pairwise comparisons showed not statistical significance differences between the season 1 and season 2, *t*(17) = 1.44, p = 0.16, d = 0.41. However, revealed significant differences between the ranking group 1 and the ranking group 3, *t*(17) = 2.92, p = 0.009, d = 0.78. The comparison between ranking group 2 and ranking group 3, *t*(17) = 1.21, *p* = 0.24, d = 0.26, did not reveal statistical significance (Fig. 15). In the same line of previous analysis, a two-way mixed ANOVA with ranking group means ball loss per match reflected a significant main effect of season condition, F (2.4) = 6.53, p = 0.003, η^2^ = 0.79. Data showed that in values of season 1 were lower than in season 2 and 3. In addition, data of season 2 were higher than in season 3. The main effect of ranking group condition and interaction between ranking group and season was not significant, *F* < 1. In a reference to main effect of season condition, pairwise comparisons showed significant differences between the season 1 and season 3, *t*(17) = 3.05, p = 0.001, d = 1.10, and between the season 2 and season 3, *t*(17) = 4.08, p = 0.001, d = 1.12. The comparison between season 1 and season 2, *t*(17) = 0.71, p = 0.48, d = 0.24, did not produce statistical differences (Fig. 16). Technical data of the player during the match [Key pass per match, long pass per match, passes to the third zone per game (mean and SE)] as a function of ranking group and season condition are shown Table 3.

**Table 3:** Technical data of the player during the match [Key pass per match, long pass per match, Passes to the third zone per game (mean and SE)] as a function of Ranking group and Season Condition.

Season 1						Season 2					Season 3				
R	T	Key pass per match	Long pass per match	Passes to the third zone per game	Ball loss per match	T	Key pass per match	Long pass per match	Passes to the third zone per game	Ball loss per match	T	Key pass per match	Long pass per match	Passes to the third zone per game	Ball loss per match
**Ranking group 1**															
1	B	3.07	37.65	64.53	96.67	B	2.67	42.43	61.95	95.43	G	2.64	38.6	56.21	96.71
2	F	3.18	39.9	61.89	100.35	Ba	2.59	41.43	58.48	103.23	F	2.75	39.48	58.42	105.01
3	Ko	2.06	44.47	59.79	107.06	F	2.17	38.64	59.63	99.71	Ba	2.23	42.87	62.71	99.05
4	Ba	1.99	38.87	57.65	103.07	G	2.29	38.49	67.72	99.82	B	2.99	41.91	64.02	95.79
5	Os	2.15	42.01	56.37	107.86	An	2.04	39.37	50.77	97.67	T	2.32	37.92	54.59	97.54
6	G	2.71	38.98	65.65	93.24	T	2.32	39.81	57.12	103.57	Gö	2.02	50.05	52.21	99.97
		2.53 ± 0.53	40.31 ± 2.50	60.98 ± 3.71	101.38 ± 5.77		2.35 ± 0.24	40.03 ± 1.58	59.28 ± 5.59	99.91 ± 3.15		2.49 ± 0.36	41.81 ± 4.47	58.03 ± 4.62	99.01 ± 3.31
**Ranking group 2**															
7	K	2.17	41.53	59.16	104.76	A	1.71	44.86	47.22	104.82	S	2.47	42.63	55.08	95.98
8	A	1.50	44.63	54.5	112.99	Ge	2.41	42.38	52.97	102.53	K	1.83	39.43	48.74	93.75
9	An	1.81	35.45	53.67	97.13	Ko	1.93	47.01	63.33	103.98	Ka	1.66	43.12	53.08	96.33
10	Ge	1.76	38.92	54.76	104.05	K	1.91	37.68	52.79	93.65	M	1.76	45.99	50.03	93.08
11	Bu	2.03	42.24	59.32	104.26	Kar	1.98	45.17	57.59	107.04	A	1.67	45.66	53.11	99.42
12	T	2.29	59.01	56.5	96.52	Al	2.38	37.02	52.26	101.98	Al	2.57	41.28	49.93	96.03
		1.93 ± 0.29	43.63 ± 8.16	56.32 ± 2.44	103.29 ± 6.03		2.05 ± 0.28	42.35 ± 4.15	54.36 ± 5.49	102.33 ± 4.62		1.99 ± 0.41	43.02 2.52	51.66 ± 2.45	95.77 ± 2.24
**Ranking group 3**															
13	R	2.04	36.06	50.16	96.03	O	2.82	41.5	55.98	98.24	Bu	1.62	41.69	52.46	96.12
14	Ga	–				B	1.91	39.23	53.59	105.78	An	1.89	43.38	56.67	94.46
15	Ka	2.26	35.76	54.18	96.77	Ka	1.99	37.84	50.99	99.99	Ko	1.89	41.17	54.19	95.88
16	S	2.49	44.13	57.99	107.6	R	2.36	43.08	53.33	97.86	O	1.79	39.76	49.97	93.26
17	E	1.71	40.3	53.03	99.74	Ga	–	–	53.37	–	Ge	1.70	45.07	51.09	101.41
18	M	1.22	44.47	55.41	107.25	Ad	1.13	44.19	47.8	101.45	Kar	1.52	44.06	51.29	100.27
		1.94 ± 0.50	40.14 ± 4.20	54.15 ± 2.89	101.48 ± 5.60		2.04 ± 0.62	41.17 ± 2.63	52.51 ± 2.80	100.66 ± 3.20		1.74 ± 0.15	42.52 ± 1.99	52.61 ± 2.45	96.90 ± 3.24

Data are presented as means and standard deviations. TD: Total Distance; THID: Total high intensity running distance; TS: Total number of sprints; TSD: Total sprint distance; R: Ranking; Teams: Beşiktaş (B); Fenerbahçe (F); Konyasport (Ko); Başakşehir (Ba); Osmanlıspor (O); Galatasaray (G); Kasımpaşa (K); Akhisar (A); Antalyaspor (An); Gençlerbirliği (Ge); Bursaspor (Bu); Trabzonspor (T); Rizespor (R); Gaziantepspor (Ga); Kayserispor (Ka); Sivasspor (S); Eskişehirspor (E); Mersin İY (M); Karabükspor (Kar); Alanyaspor (Al); Adanaspor (Ad). Göztepe (Gö). and Malatyaspor (M).

**Figure 17:**
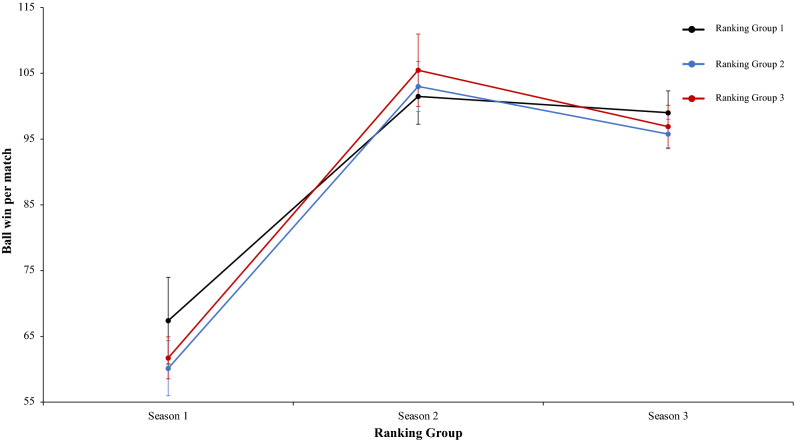
Technical performance. Mean mean ball win per match (± SE) as a funtion of Ranking and Season Condition.

**Figure 18:**
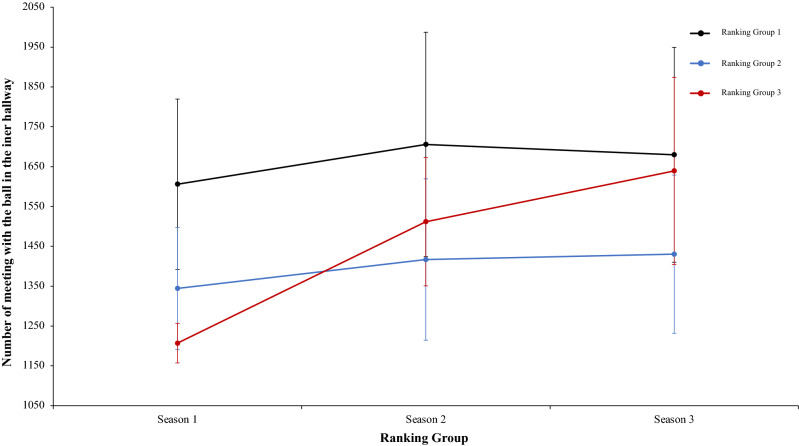
Technical performance. Mean number of meeting with the ball in the inner hallway (± SE) as a funtion of Ranking and Season Condition.

**Figure 19:**
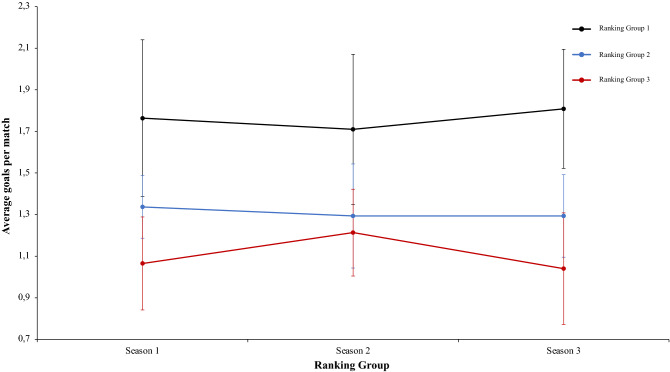
Technical performance. Mean average goals per match (± SE) as a funtion of Ranking and Season Condition.

**Figure 20:**
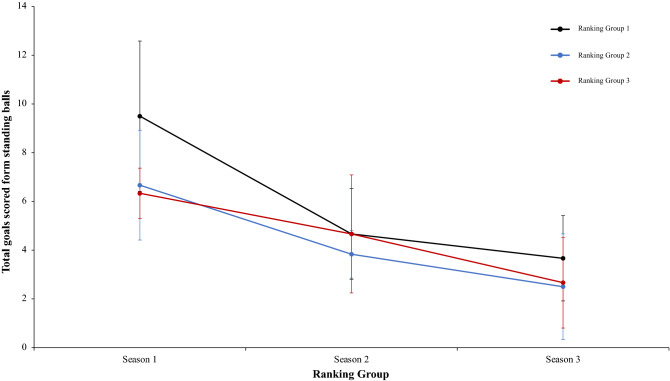
Technical performance. Mean total goals scored form standing balls (± SE) as a funtion of Ranking and Season Condition.

A two-way mixed ANOVA with ranking group means ball win per match reflected a significant main effect of season condition, F (2.4) = 475.66, p = 0.001, η^2^ = 0.98. Data showed that in values of season 1 were lower than in season 2 and 3. In addition, data of season 2 were higher than in season 3. The main effect of ranking group condition and interaction between ranking group and season condition did not reflect significative differences, *F* < 1, and F (2.4) = 2.44, p = 0.06, η^2^ = 0.17, respectively. In a reference to main effect of season condition, pairwise comparisons showed significant differences between the season 1 and season 2, *t*(17) = − 21.42, p = 0.001, d = − 8.77, and between the season 1 and season 3, *t*(17) = − 23.24, p = 0.001, d = − 8.77. The comparison between season 2 and season 3, *t*(17) = 4.98, p = 0.41, d = 1.60, did not reveal statistical significant differences (Fig. 17). As the previous analysis, a two-way mixed ANOVA with ranking group means number of meetings with the ball in the inner hallway, revealed a significant main effect of season condition, F (2.4) = 4.60, p = 0.015, η^2^ = 0.60. The values of season 1 were lower than in season 2 and 3. The main effect of ranking group condition and the interaction between ranking group and season was not significant, F (2.4) = 5.58, p = 0.07, η^2^ = 0.73, and F (2.4) = 1.49, p = 0.22, η^2^ = 0.11, respectively. In connection to main effect of season condition, pairwise comparisons showed significant differences between the season 1 and the season 2, *t*(17) = − 2.71, p = 0.01, d = − 0.87, and between season 1 and season 3, *t*(17) = − 2.79, p = 0.01, d = − 0.99. Finally, the comparison between season 2 and season 3, *t*(17) = − 1.17, p = 0.25, d = − 0.16, was not significant (Fig. 18). Furthermore, a two-way mixed ANOVA with ranking group means average goals per match, revealed a significant main effect of ranking group condition, F (2.4) = 59.45, p = 0.001, η^2^ = 0.96. teams of ranking group 1 have an average goals per match generally higher than ranking group 2 or 3. Pairwise comparisons showed significant differences between the ranking group 1 and the ranking group 2, *t*(17) = 4.60, p = 0.001, d = 1.61, and between the ranking group 1 and ranking group 3, *t*(17) = 10.30, p = 0.001, d = − 2.40. The comparison between ranking group 2 and ranking group 3, showed significant differences, *t*(17) = 2.85, p = 0.01, d = 1.14. Finally, the main effect of season condition and the interaction between ranking group and season condition did not reveal significant differences, *F* < 1 in both cases (Fig. 19). A two-way mixed ANOVA with ranking group means total goals scored from standing balls, showed a significant main effect of season condition, F (2.4) = 23.62, p = 0.001, η^2^ = 0.92. The values of season 1 were higher than in Season 2 and 3. Pairwise comparisons showed significant differences between the season 1 and season 2, *t*(17) = 4.38, p = 0.001, d = 1.59, and between the season 1 and season 3, *t*(17) = 6.97, p = 0.001, d = 2.24. Also, the comparison between season 2 and season 3, showed significant differences, *t*(17) = 2.65, p = 0.01, d = 0.78. The main effect of Group Condition and the interaction between ranking group and Season were not significant, F (2.4) = 3.33, p = 0.14, η^2^ = 0.62, and, *F* < 1, respectively. (Fig. 20). Technical data of the player during the match [Ball win per match. Number of meetings with the ball in the inner hallway. Average goals per game and Total goal scored from standing balls (mean and SE)] as a function of ranking group and season condition are shown Table 4.

**Table 4:** Technical data of the player during the match [Ball win per match. Number of meeting with the ball in the inner hallway.

Season 1						Season 2					Season 3				
R	T	Ball win per match	N. of meetings with the ball in the inner hallway	Average goals per game	Total goal scored from standing balls	T	Ball win per match	N. of meetings with the ball in the inner hallway	Average goals per game	Total goal scored from standing balls	T	Ball win per match	N. of meetings with the ball in the inner hallway	Average goals per game	Total goal scored from standing balls
**Ranking group 1**															
1	B	61.88	1650	2.35	8	B	95.6765	1848	2.14	3	G	96.71	1729	2.06	4
2	F	67.38	1514	1.75	10	Ba	100.5882	1661	1.85	7	F	105.01	1634	2.13	5
3	Ko	73.88	1514	1.29	8	F	101	1362	1.81	2	Ba	99.05	1298	1.71	1
4	Ba	71.68	1610	1.63	15	G	100.2647	2136	1.91	5	B	95.79	2111	1.89	6
5	Os	72.24	1357	1.53	6	An	108.6471	1449	1.37	6	T	97.54	1539	1.72	3
6	G	57.26	1990	2.03	10	T	102.7353	1778	1.18	5	Gö	99.97	1767	1.34	3
		67.39 ± 6.59	1605.83 ± 213.70	1.76 ± 0.38	9.50 ± 3.08		101.49 ± 4.22	1705.67 ± 281.66	1.71 ± 0.36	4.67 ± 1.86		99.01 ± 3.31	1679.67 ± 269.69	1.81 ± 0.29	3.67 ± 1.75
**Ranking group 2**															
7	K	61.18	1288	1.46	4	A	101.4706	1161	1.41	3	S	95.98	1116	1.23	5
8	A	67.82	1228	1.23	5	Ge	101.6471	1231	0.97	4	K	93.75	1301	1.56	3
9	An	59.88	1313	1.55	8	Ko	98.6176	1679	1.17	3	Ka	96.33	1589	1.2	1
10	Ge	57.06	1175	1.23	9	K	100.9118	1573	1.36	5	M	93.08	1603	1.05	5
11	Bu	58.85	1506	1.38	5	Kar	108.1765	1504	1.16	5	A	99.42	1587	1.21	1
12	T	56.09	1556	1.17	9	Al	107.2059	1353	1.69	3	Al	96.03	1386	1.51	0
		60.15 ± 4.19	1344.33 ± 153.17	1.34 ± 0.15	6.67 ± 2.25		103.00 ± 3.80	1416.83 ± 202.37	1.29 ± 0.25	3.83 ± 0.98		95.77 ± 2.24	1430.33 ± 198.51	1.29 ± 0.20	2.50 ± 2.17
**Ranking group 3**															
13	R	60.68	1130	1.17	6	O	113.6176	1650	1.09	3	Bu	96.12	1987	1.18	5
14	Ga	64.97	1206		6	B	97.7059	1490	1.03	3	An	94.46	1345	1.1	0
15	Ka	58.76	1220	0.73	7	Ka	103.8235	1584	1.41	6	Ko	95.88	1565	1.04	1
16	S	64.59	1195	1.03	5	R	104.9118	1303	1.38	4	O	93.26	1678	1.35	4
17	E	57.5	1285	1.13	6	Ga	109.5588	1347	–	9	Ge	101.41	1456	1.02	3
18	M	63.85	1207	0.94	8	Ad	103.2353	1696	0.96	3	Kar	100.27	1804	0.55	3
		61.73 ± 3.19	1207.17 ± 49.65	1.00 ± 0.18	6.33 ± 1.03		105.48 ± 5.50	1511.67 ± 160.92	1.17 ± 0.21	4.67 ± 2.42		96.90 ± 3.24	1639.17 ± 234.63	1.04 ± 0.27	2.67 ± 1.86

Average goals per game and Total goal scored from standing balls (mean and SE)] as a function of Ranking group and Season Condition.

Data are presented as means and standard deviations. TD: Total Distance; THID: Total high intensity running distance; TS: Total number of sprints; TSD: Total sprint distance; R: Ranking; Teams: Beşiktaş (B); Fenerbahçe (F); Konyasport (Ko); Başakşehir (Ba); Osmanlıspor (O); Galatasaray (G); Kasımpaşa (K); Akhisar (A); Antalyaspor (An); Gençlerbirliği (Ge); Bursaspor (Bu); Trabzonspor (T); Rizespor (R); Gaziantepspor (Ga); Kayserispor (Ka); Sivasspor (S); Eskişehirspor (E); Mersin İY (M); Karabükspor (Kar); Alanyaspor (Al); Adanaspor (Ad). Göztepe (Gö). and Malatyaspor (M).

**Figure 21:**
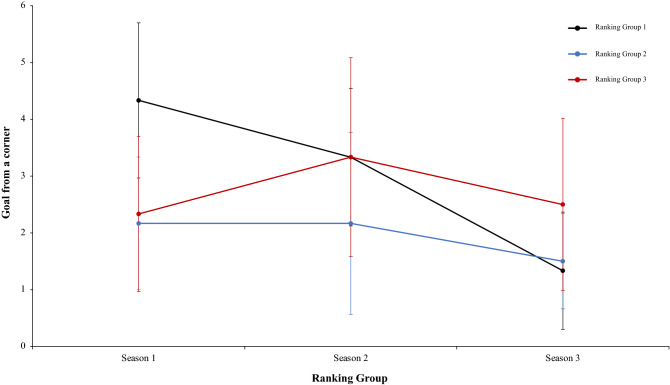
Technical performance. Mean total goal forma a corner (± SE) as a funtion of Ranking and Season Condition.

**Figure 22:**
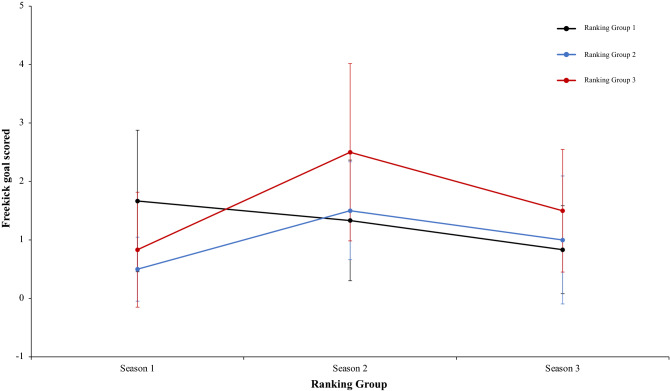
Technical performance. Mean freekick goal scored (± SE) as a funtion of Ranking and Ranking Condition.

**Figure 23:**
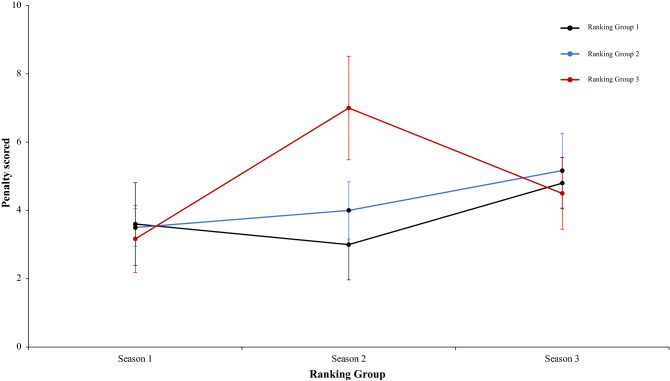
Technical performance. Mean penalty scored (± SE) as a funtion of Ranking and Season Conditon.

Continuing with the same analysis of present work, a two-way mixed ANOVA with ranking group means total goal from a corner, revealed a significant main effect of season condition, F (2.4) = 4.51, p = 0.001, η^2^ = 0.49. However, main effect of ranking group condition, F (2.4) = 1.27, p = 0.37, η^2^ = 0.38, and interaction between ranking group and season condition, F (2.4) = 2.33, p = 0.07, η^2^ = 0.17, were not significant (Fig. 21). In a reference to main effect of season condition, pairwise comparisons not showed significant differences between the season 1 and season 2, *t*(17) = 0.00, p = 1, d = 0. However, data revealed significant differences between the season 1 and season 3, and between season 2 and 3, *t*(17) = 2.54, p = 0.02, d = 0.82, and (17) = 2.62, p = 0.01, d = 0.83, respectively. Another two-way mixed ANOVA with ranking group means freekick goal scored did not reveal any effect or interaction significative, thus, the main effect of season condition, the interaction between ranking group and season and the ranking group showed: *F* < 1, (2.4) = 2.96, p = 0.07, η^2^ = 0.46, and F (2.4) = 1.67, p = 0.17, η^2^ = 0.12, *F* < 1, respectively. (Fig. 22). In this respect, a new two-way mixed ANOVA with ranking group means penalty scored did not showed significant main effects of ranking group condition, *F* < 1, and in neither in the season condition, F (2.4) = 4.00, p = 0.06, η^2^ = 0.29. However, data revealed an interaction between ranking group and season, F (2.4) = 3.53, p = 0.01, η^2^ = 0.23. (Fig. 23). Last, a two-way mixed ANOVA with ranking group means freekick goal scored from throw-in showed not significant differences in main effect of ranking group condition, (2.4) = 4.00, p = 0.11, η^2^ = 0.66, and neither in the season condition and the interaction between ranking group and season, *F* < 1. Technical data of the player during the match [Goal from a corner. Freekick goal scored. Penalty scored and Goal scored from throw-in (mean and SE)] as a function of ranking group and season condition are shown Table 5.

**Table 5:** Technical data of the player during the match [Goal from a corner. Freekick goal scored. Penalty scored and Goal scored from throw-in (mean and SE)] as a function of Ranking group and Season Condition.

Season 1						Season 2					Season 3				
R	T	Goal from a corner	Freekick goal scored	Penalty scored	Goal scored from throw-in	T	Goal from a corner	Freekick goal scored	Penalty scored	Goal scored from throw-in	T	Goal from a corner	Freekick goal scored	Penalty scored	Goal scored from throw-in
**Ranking group 1**															
1	B	4	3	3	0.00	B	2	0	2	0.00	G	0	1	4	0.00
2	F	3	3	6	0.00	Ba	4	1	4	0.00	F	1	2	7	0.00
3	Ko	4	2	3	1.00	F	2	1	5	1.00	Ba	1	0	3	0.00
4	Ba	7	1	5	0.00	G	3	2	4	0.00	B	2	0	4	0.00
5	Os	4	0	1	0.00	An	5	1	0	0.00	T	1	1	6	0.00
6	G	4	1	9	0.00	T	4	3	2	0.00	Gö	3	1	8	0.00
		4.33 ± 1.37	1.67 ± 1.21	4.50 ± 2.81	0.17 ± 0.41		3.33 ± 1.21	1.33 ± 1.03	2.83 ± 1.83	0.17 ± 0.41		1.33 ± 1.03	0.83 ± 0.75	5.33 ± 1.97	0.00 ± 0.00
**Ranking group 2**															
7	K	1	1	1	0.00	A	1	1	3	0.00	S	1	1	4	0.00
8	A	2	1	1	0.00	Ge	2	2	7	0.00	K	2	3	5	0.00
9	An	3	0	4	0.00	Ko	0	3	3	0.00	Ka	3	1	4	0.00
10	Ge	4	0	4	0.00	K	4	1	1	0.00	M	1	1	4	0.00
11	Bu	2	0	2	0.00	Kar	4	1	8	0.00	A	1	0	6	0.00
12	T	1	1	4	0.00	Al	2	1	9	0.00	Al	1	0	6	0.00
		2.17 ± 1.17	0.50 ± 0.55	2.67 ± 1.51	0.00 ± 0.00		2.17 ± 1.60	1.50 ± 0.84	5.17 ± 3.25	0.00 ± 0.00		1.50 ± 0.84	1.00 ± 1.10	4.83 ± 0.98	0.00 ± 0.00
**Ranking group 3**															
13	R	2	0	3	0.00	O	2	2	6	0.00	Bu	2	0	3	0.00
14	Ga	2	1	2	0.00	B	2	0	5	0.00	An	0	1	3	0.00
15	Ka	3	2	3	0.00	Ka	5	4	11	0.00	Ko	4	3	6	0.00
16	S	3	0	2	0.00	R	6	2	5	0.00	O	2	1	7	0.00
17	E	0	2	5	0.00	Ga	3	3	6	0.00	Ge	3	2	2	0.00
18	M	4	0	3	0.00	Ad	2	4	8	0.00	Kar	4	2	4	0.00
		2.33 ± 1.37	0.83 ± 0.98	3.00 ± 1.10	0.00 ± 0.00		3.33 ± 1.75	2.50 ± 1.52	6.83 ± 2.32	0.00 ± 0.00		2.50 ± 1.52	1.50 ± 1.05	4.17 ± 1.94	0.00 ± 0.00

Data are presented as means and standard deviations. TD: Total Distance; THID: Total high intensity running distance; TS: Total number of sprints; TSD: Total sprint distance; R: Ranking; Teams: Beşiktaş (B); Fenerbahçe (F); Konyasport (Ko); Başakşehir (Ba); Osmanlıspor (O); Galatasaray (G); Kasımpaşa (K); Akhisar (A); Antalyaspor (An); Gençlerbirliği (Ge); Bursaspor (Bu); Trabzonspor (T); Rizespor (R); Gaziantepspor (Ga); Kayserispor (Ka); Sivasspor (S); Eskişehirspor (E); Mersin İY (M); Karabükspor (Kar); Alanyaspor (Al); Adanaspor (Ad). Göztepe (Gö). and Malatyaspor (M).

**Figure 24:**
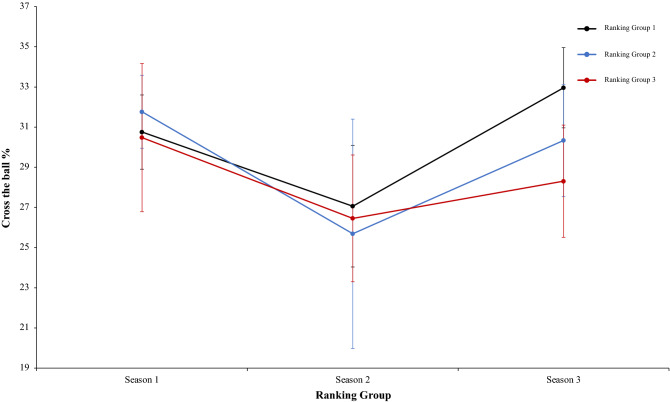
Technical performance. Mean cross the ball percentage (± SE) as a funtion of Ranking and Season Condition.

**Figure 25:**
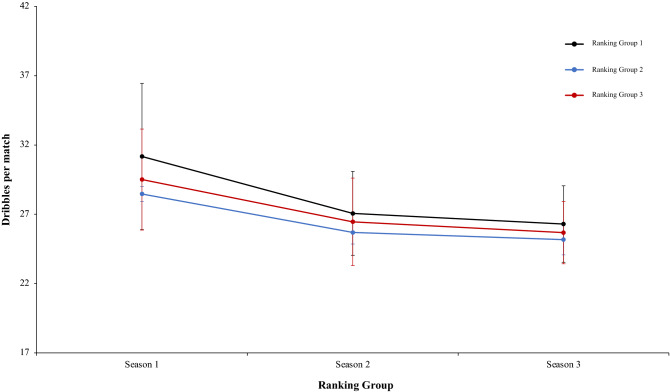
Technical performance. Mean dribbling per match (± SE) as a funtion of Group and Season Condition.

**Figure 26:**
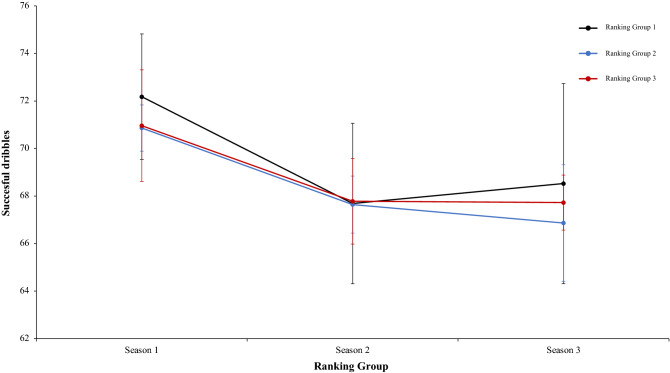
Technical performance. Means dribbling per match percentage (± SE) as a funtion of Group and Season Condition.

Finally, another two-way mixed ANOVA with ranking group means cross the ball %, revealed a significant main effect of season condition, F (2.4) = 11.31, p = 0.001, η^2^ = 0.83. In season 1 and 3 the values were higher than in Season 2. Pairwise comparisons showed significant differences between the season 1 and season 2, *t*(17) = 3.94, p = 0.001, d = 1.33, and, between season 2 and season 3, *t*(17) = − 3.78, p = 0.001, d = 0.16. In this line, comparison between season 1 and 3 did not reveal significant differences, *t*(17) = 0.53 p = 0.59, d = − 1.21. The main effect of ranking group condition and the interaction between ranking group and season condition were not significant, F (2.4) = 1.33, p = 0.35, η^2^ = 0.40, and F (2.4) = 1.12, p = 0.35, η^2^ = 0.09 (Fig. 24). Another two-way mixed ANOVA with ranking group means dribbles per match revealed a significant main effect of ranking group condition, F (2.4) = 11.74, p = 0.02, η^2^ = 0.85. Data of ranking group 1 were higher than in ranking group 2 and 3. Pairwise comparisons did not show significant differences between ranking group 1 and ranking group 2, *t*(17) = 3.15, p = 0.006, d = 0.39, neither comparison between ranking group 1 and ranking group 3, *t*(17) = 0.90, *p* = 37, d = 0.25, and, ranking group 2 and ranking group 3, *t*(17) =  − 0.52, *p* = − 0.60, d = − 0.19. In addition, data revealed a significant main effect of season condition, F (2.4) = 5.57, p = 0.006, η^2^ = 0.97. Data of season 1 were higher than in season 3. Pairwise comparisons showed significant differences between season 1 and season 3, *t*(17) = 3.15, p = 0.006, d = 0.29, Comparison between season 1 and 2, and, season 2 and 3, was not significant, *t*(17) =  − 1.77, p = 0.09, d = 0.41, and, *t*(17) = 0.53, p = 0.59, d = 0.10, respectively. The interaction between ranking group and season condition were not significant, *F* < 1 in both cases (Fig. 25). Lastly, a two-way mixed ANOVA with ranking group means successful dribbles per match percentage, revealed a significant main effect of season condition, F (2.4) = 13.03, p = 0.001, η^2^ = 0.96. In. fact, data of season 1 were higher than in Season 2 and 3. Pairwise comparisons showed significant differences between the season 1 and season 2, *t*(17) = 5.25, p = 0.001, d = 1.68, and, between the season 1 and season 3, *t*(17) = 4.17, p = 0.001, d = 1.52. In addition, comparison between season 2 and 3, did not reveal significant differences, *t*(17) = 0.01, p = 0.98, d = 0.004. The main effect of ranking group condition, F (2.4) = 3.44, p = 0.13, η^2^ = 0.63, and the interaction between ranking group and season were not significant, *F* < 1 in both cases (Fig. 26). Technical data of the player during the match [Cross the ball%. Dribbling per match and dribbling per match % (mean and SE)] as a function of ranking group and season condition are Table 6.

**Table 6:** Technical data of the player during the match [Cross the ball%. Dribbling per match and Dribbling per match % (mean and SE)] as a function of Ranking group and Season Condition.

Season 1						Season 2			Season 3				
R	T	Cross the ball %	Dribling per match	Dribling per match %	T	Goal scored from throw-in	Cross the ball %	Dribling per match	T	Goal scored from throw-in		Cross the ball %	Dribling per match
**Ranking group 1**													
1	B	29.5	35.74	76.36	B	29.49	29.49	73.13	G	30.39		26.66	69.04
2	F	33.69	35.55	71.29	Ba	22.96	22.96	63.76	F	34.06		26.08	72.31
3	Ko	29.92	23.38	68.3	F	30.52	30.52	66.6	Ba	31.81		24.76	68
4	Ba	28.48	29.94	71.47	G	28.32	28.32	69.67	B	35.29		31.57	73.76
5	Os	31.55	35.48	72.47	An	23.97	23.97	67.88	T	34.74		24.54	65.7
6	G	31.38	26.91	73.19	T	27.11	27.11	65.07	Gö	31.47		24.15	62.33
		30.75 ± 1.85	31.17 ± 5.27	72.18 ± 2.64		27.06 ± 3.03	27.06 ± 3.03	67.69 ± 1.20		32.06 ± 2.00		26.29 ± 2.76	68.52 ± 4.21
**Ranking group 2**													
7	K	31.87	32.3	71.53	A	20.39	20.39	68.47	S	31.09		20.39	66.28
8	A	33.33	24.34	70.93	Ge	30.35	30.35	67.44	K	33.07		30.35	64.25
9	An	30.5	30.43	71.4	Ko	18.18	18.18	65.65	Ka	27.79		18.18	65.37
10	Ge	29.62	28.13	69.7	K	25.95	25.95	67.54	M	27.45		25.95	68.36
11	Bu	34.42	25.21	69.66	Kar	26.05	26.05	67.52	A	28.63		26.05	65.88
12	T	30.86	30.36	71.95	Al	33.22	33.22	69.22	Al	34		33.22	71.04
		31.77 ± 1.82	28.46 ± 3.16	70.86 ± 0.97		25.69 ± 5.71	25.69 ± 5.71	67.64 ± 1.20		30.34 ± 2.80		25.69 ± 5.71	66.86 ± 2.45
**Ranking group 3**													
13	R	35.12	23.7	71.52	O	31.91	31.91	69.85	Bu	26.74		31.91	68.11
14	Ga	–	–	–	B	25.36	25.36	67.74	An	31.32		25.36	66.28
15	Ka	25.51	33.01	70.72	Ka	27.15	27.15	69.18	Ko	27.43		27.15	69.41
16	S	33.52	31.76	74.16	R	25.67	25.67	64.63	O	26.03		25.67	68.04
17	E	26.98	32.18	71.17	Ga	–	–	–	Ge	25.99		-	67.99
18	M	30.75	26.67	66.86	Ad	22.24	22.24	67.58	Kar	32.33		22.24	66.52
		30.38 ± 4.12	29.46 ± 4.07	70.89 ± 2.62		26.47 ± 3.53	26.47 ± 3.53	67.80 ± 2.01		28.31 ± 2.79		25.68 ± 2.25	67.73 ± 1.16

R: Ranking; Teams: Beşiktaş (B); Fenerbahçe (F); Konyasport (Ko); Başakşehir (Ba); Osmanlıspor (O); Galatasaray (G); Kasımpaşa (K); Akhisar (A); Antalyaspor (An); Gençlerbirliği (Ge); Bursaspor (Bu); Trabzonspor (T); Rizespor (R); Gaziantepspor (Ga); Kayserispor (Ka); Sivasspor (S); Eskişehirspor (E); Mersin İY (M); Karabükspor (Kar); Alanyaspor (Al); Adanaspor (Ad). Göztepe (Gö). and Malatyaspor (M).

## Discussion

This study aimed to analyze the seasonal variations in the physical and technical demands of Turkish Super League teams considering the teams' statuses (i.e., whether they were in the first, second, or third groups in the league) in the final rankings over three consecutive seasons. The large database and the absence of previous studies regarding the Turkish Super League are the main strengths of the current study.

Our results did not indicate an evolutionary trend in physical demands over consecutive seasons, and differently ranked teams presented similar physical responses. On the other hand, evolutionary trends were observed concerning technical variables. Specifically, the number of lost balls, ball touches in the central corridor, and goals from set pieces increased from season one to the others, while the number of successful dribbles reduced over time. Finally, rank-based differences were observed in technical parameters. Top teams presented a higher percentage of successful passes, longer ball possession, more passes per minute, more key passes, and more passes to the pitch's final third (the pitch zone closest to the opponent's goal).

We did not find an evolutionary tendency in players' physical responses over seasons. While some previous studies found an increase in high-intensity running and sprinting demands over consecutive seasons in the English Premier League^12,21^, the Spanish La Liga^22^, and in the Chinese Super League^23^, others reported few changes in physical parameters over consecutive seasons^24^. Interestingly, the studies that showed differences include data from seasons played up until 2013, while the most recent study^24^ (including the current database) showed no increases in physical performance over the years. In the study in the Chinese Super League^23^, more prominent differences were observed when the latest monitored seasons (2017 and 2013) were compared. For this reason, we argue that although evolutions in physical performance were achieved at the beginning of the decade, similar evolutions might not be observed in current matchs. Therefore, technological and theoretical advances (such as the availability of load management tools like GPS devices) that have occurred in recent years have enhanced players' training and increased match-related physical performance but are not able to go further. As previous studies focused mainly on top leagues, such as the Premier League^12,21^ and La Liga^24^, specific characteristics of the Turkish national league as an emergent competition might be taken into account to interpret the current results.

Although the physical performance did not evolve over the years, changes in technical performance were observed. The teams tended to get better at maintaining ball possession and finding goal-scoring opportunities because the number of lost balls decreased and the ability to keep the possession in the central corridor increased. The literature shows that passing performance increases over the years in top^11,21,25^ and emergent^23^ national soccer leagues. The successful Pep's Barcelona influenced coaches across the world with their ball-possession-based offensive strategy^26^, which might explain the evolutionary tendency of increases in the number of passes and passing accuracy.

Interestingly, this tendency was also observed when comparing groups against each other, with top-ranked teams showing better ball possession, passes per minute, and passing accuracy than bottom-ranked teams. The current results regarding differences between top- and bottom-ranked teams are similar to the literature^27^, although the current results must not be understood as a one-size-fits-all recommendation. This is because, in the case of the Turkey Super League, adopting a ball-possession offensive strategy appears to be related to successful performance. We strongly recommend enlarging the sample and the number of countries investigated to support such an assumption. On the other hand, we did not find any group-specific evolutions as Bradley et al.^11^ did. This finding suggests that evolutionary tendencies were similar across groups and that group-related differences in game style are likely to be stable over several years.

The current study has some limitations. First, data were collected from only three consecutive seasons. Future studies are recommended to enlarge the database. Such studies might be more suitable for detecting evolutionary trends in match-related variables. Also, we were not able to account for the tactical aspects of the game because it was not possible to include tactical-related variables based on the available data. As previous studies showed differences in tactical performance over the years^28^, we recommend future studies to include positional and observational data related to players' and teams' tactical performances to better understand evolutionary tendencies in match-related performances in elite soccer.

From a practical point of view, coaches and clubs might benefit from the information obtained in this study in two points. Firstly, the ball-possession strategy tendency observed in the Turkish League seems to indicate that players' training should be adapted to the new requirements of the game. At this point, including game-based possession drills (such as the well-known "rondos") seems interesting to allow the players to adapt to the game flow and requirements. Secondly, as an evolutionary trend was observed, clubs and coaches should always account for the possible changes experienced in the game. To enhance the training specificity to the requirements of the constantly evolved game, investing in match analysis departments seems mandatory. By this, clubs will get up-to-date information that will allow them to adapt training programs and enchance the players’ development constantly.

## Conclusions

In summary, our findings did not show an evolutionary trend in physical demands in consecutive seasons, and the teams that were ranked differently gave similar physical responses. On the other hand, evolutionary trends regarding technical variables were observed. Specifically, the numbers of lost balls, ball touches to the middle lane, and goals from sets increased from one season to the next, while the number of successful dribbles decreased over time. Finally, sequence-based differences were observed in technical parameters. The top teams were better in terms of successful pass percentage, ball possession, passes per minute, and passes to the last third of the pitch.

## Data Availability

The datasets generated during and analyzed during the current study are available from the corresponding author on reasonable request.

## References

[1] Rein R, Memmert D (2016). Big data and tactical analysis in elite soccer: Future challenges and opportunities for sports science. Springerplus.

[2] Trecroci A, Boccolini G, Duca M, Formenti D, Alberti G (2020). Mental fatigue impairs physical activity, technical and decision-making performance during small-sided games. PLoS ONE.

[3] Sarmento H, Clemente FM, Araújo D, Davids K, McRobert A, Figueiredo A (2018). What performance analysts need to know about research trends in association football (2012–2016): A systematic review. Sport. Med..

[4] Sarmento H (2014). Match analysis in football: a systematic review. J. Sports Sci..

[5] Carling C, Bloomfield J, Nelsen L, Reilly T (2008). The role of motion analysis in elite soccer. Sport. Med..

[6] PalucciVieira LH, Carling C, Barbieri FA, Aquino R, Santiago PRP (2019). Match running performance in young soccer players: A systematic review. Sports Med..

[7] Low B (2020). A systematic review of collective tactical behaviours in football using positional data. Sports Med..

[8] Memmert D, Lemmink KAPM, Sampaio J (2017). Current approaches to tactical performance analyses in soccer using position data. Sport. Med..

[9] Aquino R (2018). Influence of situational variables, team formation, and playing position on match running performance and social network analysis in Brazilian professional soccer players. J. Strength Cond. Res..

[10] Castellano J, Blanco-Villaseñor A, Álvarez D (2011). Contextual variables and time-motion analysis in soccer. Int. J. Sports Med..

[11] Bradley PS (2016). Group-specific evolution of match performance characteristics in the English Premier League: It’s getting tougher at the top. J. Sports Sci..

[12] Barnes C, Archer DT, Hogg B, Bush M, Bradley PS (2014). The evolution of physical and technical performance parameters in the english premier league. Int. J. Sports Med..

[13] Varley MC (2017). Physical and technical performance of elite youth soccer players during international tournaments: Influence of playing position and team success and opponent quality. Sci. Med. Footb..

[14] Wallace JL, Norton KI (2014). Evolution of World Cup soccer final games 1966–2010: Game structure, speed and play patterns. J. Sci. Med. Sport.

[15] Cohen J (1992). A power primer. Psychol. Bull..

[16] Sawilowsky SS (2009). Very large and huge effect sizes. J. Mod. Appl. Stat. Methods.

[17] Baysal, S., Duygulu, P. & Kayalar, C. Çoklu sabit kamera sistemleri ve futbol sahasi modeli arasindaki homografi üzerine bir tartişma. In *2012 20th Signal Processing and Communications Applications Conference, SIU 2012, Proceedings* (2012). 10.1109/SIU.2012.6204759.

[18] Baysal S, Duygulu P (2016). Sentioscope: A soccer player tracking system using model field particles. IEEE Trans. Circuits Syst. Video Technol..

[19] Baysal S (2016). Model Field Particles with Positional Appearance Learning for Sports Player Tracking.

[20] Baysal S, Duygulu P (2013). A line based pose representation for human action recognition. Signal Process. Image Commun..

[21] Bush M, Barnes C, Archer DT, Hogg B, Bradley P (2015). Evolution of match performance parameters for various playing positions in the English Premier League. Hum. Mov. Sci..

[22] Alcantarilla-Pedrosa M, Álvarez-Santana D, Hernández-Sánchez S, Yañez-Álvarez A, Albornoz-Cabello M (2021). Assessment of external load during matches in two consecutive seasons using the Mediacoach^®^ video analysis system in a spanish professional soccer team: Implications for injury prevention. Int. J. Environ. Res. Public Health.

[23] Zhou C, Gómez MÁ, Lorenzo A (2020). The evolution of physical and technical performance parameters in the Chinese Soccer Super League. Biol. Sport.

[24] Pons E (2021). A longitudinal exploration of match running performance during a football match in the spanish la liga: A four-season study. Int. J. Environ. Res. Public Health.

[25] Yi Q, Liu H, Nassis GP, Gómez MÁ (2020). Evolutionary trends of players’ technical characteristics in the UEFA champions league. Front. Psychol..

[26] Buldú JM, Busquets J, Echegoyen I, Seirullo F (2019). Defining a historic football team: Using Network Science to analyze Guardiola’s F.C. Barcelona. Sci. Rep..

[27] Yi Q, Gómez MÁ, Liu H, Sampaio J (2019). Variation of match statistics and football teams’ match performance in the group stage of the UEFA champions league from 2010 to 2017. Kinesiology.

[28] Barreira D, Garganta J, Castellano J, Prudente J, Anguera MT (2014). Evolution of attacking patterns in elite-level soccer between 1982 and 2010: The application of lag sequential analysis. Rev. Psicol. del Deport..

